# Novel targeted therapies for mantle cell lymphoma

**DOI:** 10.18632/oncotarget.426

**Published:** 2012-02-22

**Authors:** Lapo Alinari, Beth Christian, Robert A. Baiocchi

**Affiliations:** ^1^ Division of Hematology, Department of Medicine, College of Medicine, The Ohio State University, Columbus, Ohio, USA

**Keywords:** mantle cell lymphoma, targeted therapy, apoptosis, autophagy, lysosomal cell death

## Abstract

Mantle cell lymphoma (MCL) is an aggressive B-cell malignancy characterized by short median survival despite intensive therapies. The clinical behavior of MCL most likely relates to the complex pathophysiology of the disease which includes its genetic hallmark, the chromosomal translocation t(11;14) resulting in aberrant expression of cyclin D1, alteration in the DNA damage response, and constitutive activation of key antiapoptotic pathways such as phosphatidyl-inositol 3-kinase (PI3K)/Akt and nuclear factor-kB (NF-kB). Together, these changes result in cell cycle dysregulation and give rise to profound genetic instability. Given this complex pathophysiology, the limited number of options for patients with relapsed/refractory MCL, and the difficulty in achieving long-lasting remissions with conventional approaches, it is essential to explore new treatment options targeting the pathophysiology of MCL. We have recently reported that milatuzumab, a fully humanized anti-CD74 monoclonal antibody (mAb), in combination with anti-CD20 mAbs has significant preclinical and clinical activity in MCL. Here we discuss these results, provide additional insights into milatuzumab-mediated MCL cell death, and report preliminary data on the activity of other targeted biologic agents including PCI-32765, CAL-101 and mammalian target of rapamycin (mTOR) inhibitors currently undergoing evaluation at our institution and others.

## INTRODUCTION

Mantle cell lymphoma (MCL) is a neoplasm classified as an aggressive B-cell malignancy [1] that accounts for approximately 3 to 8% of Non-Hodgkin's lymphoma (NHL) cases diagnosed annually [2-4]. MCL patients are typically diagnosed at age 60 to 65 years, and present with generalized non-bulky lymphadenopathy and frequent extranodal disease burden [5]. While some patients present with indolent disease, most have a more aggressive disease course, and virtually all MCL patients require systemic therapy [6-12]. Median overall survival (OS) of MCL patients has been reported to be approximately three years [4, 5]; however recent series have shown an (OS) of 5 to 7 years [13, 14]. Aggressive therapies including chemo-immunotherapy [8, 15] or high dose chemotherapy followed by autologous stem cell transplant [16, 17] have been shown to improve outcome; however, no standard therapy offers the potential for cure. The high response rate (RR) and longer progression free survival (PFS) obtained with these regimens certainly represent a major advance. However, several challenges remain in the care of patients with MCL including the absence of curative therapy, associated major toxicities, and the limited number of treatment options for patients with relapsed/refractory disease [18].

The pathobiology of MCL is complex and includes alterations in the cell cycle as a consequence of cyclin D1 over-expression driven by the chromosomal translocation t(11;14)(q13;q32) [19], abnormalities in the DNA damage response [20], and constitutive activation of key antiapoptotic pathways including phosphatidyl-inositol 3-kinase (PI3K)/Akt and nuclear factor-kB (NF-kB) [21, 22]. This biologic complexity may explain the natural history of MCL which is characterized by a course of increasingly short-lived progressive relapses [23]. Novel treatment approaches targeting MCL pathobiology are therefore essential.

Monoclonal antibodies (mAbs) targeting surface proteins and tumor cell survival pathways have become widely adopted in the treatment of patients with lymphoma for a variety of reasons. These include improvement of patient outcomes when combined with chemotherapy and limited toxicity profiles, making mAbs ideal alternative options for heavily pretreated patients with relapsed/refractory disease [24]. Rituximab (Genentech Inc, San Francisco, CA), a chimeric anti-human CD20 mAb, has been widely utilized to treat MCL patients [25, 26]. As a single agent, rituximab has been tested in untreated as well as pretreated patients with RR of approximately 30% and a median response duration of 6 months [25, 27]. In combination with anthracycline-based regimens, rituximab significantly improved RR and time to progression of MCL patients when compared to patients treated with chemotherapy alone [28]. Furthermore, a recent meta-analysis of seven randomized controlled trials indicated that rituximab plus chemotherapy may prolong OS in MCL as compared to chemotherapy alone [8]. The promising results from several clinical trials support the concept of combining mAbs to target multiple pathways in NHLs [29, 30]. Dual antibody therapy offers several advantages over a single mAb approach including potentially enhanced activity when compared to single mAb or chemotherapy approachs due to alternative mechanisms of action, lack of significant hematologic toxicities, ability to overcome single-agent resistance mechanisms, and improved tolerance in heavily pre-treated, older patients or patients with significant comorbidities.

Milatuzumab (hLL1, IMMU-115, Immunomedics Inc., Morris Plains, NJ) is a fully humanized mAb specific for CD74 [31], a type II transmembrane glycoprotein associated with MHC class II that was recently found to play an important role in the maturation and proliferation of B-cells by activating the PI3K/Akt and the NF-κβ pathways [32, 33]. CD74 is expressed on the majority of B-cell malignancies including MCL [34], making it an attractive therapeutic target. Milatuzumab demonstrated anti-proliferative activity in transformed B-cell lines and improved survival in preclinical models [33, 35]. Unlike rituximab, milatuzumab mainly causes direct cytotoxicity with little or no role for antibody dependent cell-mediated cytotoxicity (ADCC) or complement-dependent cytotoxicity (CDC) [35, 36]. Phase I testing in multiple myeloma demonstrated that milatuzumab is well-tolerated [37] and is presently being evaluated in phase I/II clinical trials for the treatment of NHL and chronic lymphocytic leukemia (NCT00868478; NCT00603668; NCT00504972).

We recently reported that the combination of milatuzumab and rituximab has preclinical *in vitro* and *in vivo* activity in MCL [34], with the combination approach being justified by the fact that these two mAbs target distinct antigens lacking known association and, as single agents, have demonstrated substantial anti-tumor activity in B cell non-Hodgkin's lymphoma (NHL) cells [35, 36]. Treatment of MCL cell lines and primary patient tumor cells with either immobilized milatuzumab or rituximab resulted in statistically significant enhanced cell death, which was further potentiated when the two mAbs were combined. We found that this combination mAb treatment induced a caspase-independent non-classical apoptotic, non-autophagic cell death pathway. Furthermore, milatuzumab- and rituximab-induced cell death was mediated by radical oxygen species (ROS) generation and loss of mitochondrial membrane potential. We also highlighted the importance of actin dynamics and disruption of the NF-κB pathway in milatuzumab- and rituximab-mediated cell death. While it is known that mAbs directed to CD20 and HLA-DR can elicit lysosome-mediated cell death [38, 39], we recently showed that milatuzumab also has the ability to induce lysosomal membrane permeabilization (LMP) (Alinari L and Baiocchi RA, unpublished data). Acridine orange (AO) at acidic pH (for example in lysosomes) fluoresces red, and when AO leaks into a neutral pH (for example in cytosol) it causes an increase in green fluorescence which was detected in milatuzumab treated MCL cells by flow cytometry. LMP is a well established mechanism of cell death [40] which happens as a consequence of the translocation of lysosomal hydrolases (such as cathepsin) from the lysosomal compartment to the cytosol. It remains to be clarified if ROS generation and loss of mitochondrial membrane potential are the triggers or occur as a consequence of LMP in milatuzumab-treated MCL cells.

We have also shown that FTY720, an immunosuppressive agent recently approved by the FDA for the treatment of relapsed multiple sclerosis [41], has significant *in vitro* activity in MCL, promoting MCL cell death through caspase-independent ROS generation and down-modulation of p-Akt and Cyclin D1, with subsequent accumulation of cells in G0/G1 and G2/M phases of the cell cycle [42]. We recently further elucidated the mechanism of action of FTY720 in MCL cell lines and showed that FTY720 treatment of MCL cells leads to autophagy blockage and LMP with subsequent translocation of lysosomal hydrolases in the cytosol [43]. FTY720 treatment of MCL cells led to increase CD74 expression by preventing its degradation in the lysosomal compartment demonstrating for the first time that a druggable target can be induced by autophagy blockade. The combination of FTY720 and milatuzumab resulted in statistically significant enhanced cell death *in vitro* and significantly prolonged survival in a mouse model of human MCL. The most clinically relevant aspects of these findings are: 1) we were able to significantly increase the level of a “druggable” target (CD74) using an active anti-MCL agent (FTY720), generating more CD74 available for milatuzumab binding, and 2) because of the FTY720 effect on CD74 expression, we were able to significantly decrease the dose of these two agents without affecting the synergistic effect on MCL cell viability, suggesting that lower dosages may be used *in vivo* resulting in a more favorable toxicity profile.

The primary toxicity of FTY720 is immunosuppression, which occurs via interaction with sphingosine 1-phosphate (S1P) receptors [41]. OSU-2S, a non-phosphorylatable FTY720 derivative recently developed at the Ohio State University [44] has similar cytotoxic activity in MCL cell lines, suggesting that the S1P signaling is not necessary for FTY720-mediated anti-tumor effect. Considering that OSU-2S is predicted to have less immunosuppressive effects as compared to FTY720, this compound may provide anti-tumor activity without the S1P-mediated immune suppressive properties. More studies are needed to fully characterize the immune modulatory and anti-tumor activity properties of OSU-2S.

In an attempt to increase the activity of a combined mAbs approach, we also tested two novel humanized anti-CD20 antibodies, veltuzumab (Immunomedics Inc.) and ofatumomab (Genmab Inc, Princeton NJ), and showed that the combination of milatuzumab with either veltuzumab or ofatumomab resulted in enhanced cell death compared to either agent alone [34]. Veltuzumab, which differs from rituximab due to an amino acid substitution in the compliment binding region of the variable heavy chain of CDR3, was designed to limit infusion reactions and reduce infusion times as compared to rituximab [45]. However, it has been reported that veltuzumab has several additional advantages over rituximab including improved CDC with equal rates of ADCC, slower off-rates, shorter infusion times, higher potency, and improved therapeutic responses in animal models [46]. Phase II clinical testing of veltuzumab demonstrated single agent activity in patients with relapsed and refractory NHL [47]. As a result of the anti-tumor activity we observed *in vitro* with combined veltuzumab and milatuzumab [34], and the promising preliminary results obtained with single agent veltuzumab in NHL patients [47, 48], we initiated a phase I/II trial testing the combination of milatuzumab and veltuzumab in patients with relapsed or refractory B-cell NHL (NCT00989586) at the Ohio State University [49]. Patients received veltuzumab 200 at mg/m^2^ weekly combined with escalating doses of milatuzumab at 8, 16, and 20 mg/kg twice per week of weeks one through four (induction), and weeks twelve, twenty, twenty-eight, and thirty-six (extended induction). The dosing schedule for this combination trial is based on the extended induction rituximab schedule used by Ghielmini and the Swiss Group for Clinical Cancer Research (SAAK) investigators [50, 51] and this dosing schedule has been adopted by the Alliance (formerly Cancer and Leukemia Group B) Cooperative Group as the backbone for studies of combined mAbs [52]. All patients received pre-medication with acetominophen, diphenhydramine, and famotidine prior to each veltuzumab dose and acetominophen, diphenhydramine, and hydrocortisone before and after each milatuzumab dose. In the first two cohorts of the phase I study, three of six patients experienced grade 3 infusion reactions during or immediately following the milatuzumab infusion. Due to the observed infusion reactions with milatuzumab, the protocol was amended to include additional premedication with intravenous antihistamine, and dexamethasone 20 mg pre-milatuzumab and 10 mg post-milatuzumab. The schedule of treatment was also modified so that the antibodies were no longer administered on the same day and milatuzumab was given once weekly. Following the modification to the protocol, no further grade 3 infusion reactions were observed. In the phase I study, at the time of last reporting, eighteen patients were enrolled and had completed at least four weeks of combined veltuzumab and milatuzumab. Histologies included follicular NHL grade 1-2 (n=5), grade 3 (n=5), transformed follicular (n=1), diffuse large B-cell lymphoma (n=4), marginal zone lymphoma (n=1), MCL (n=1), and lymphoplasmacytic lymphoma (n=1). Median age was 65 years (range 44-81), and patients received a median of 3 prior therapies (range 1 – 9), including 3 patients who had undergone prior autologous stem cell transplant. Ten of 18 (56%) patients were refractory to rituximab defined as having less than a partial response to the last rituximab-containing regimen. Other grade 3-4 toxicities at least possibly related to protocol therapy consisted of lymphopenia (n=10, 56%), fatigue (n=2, 11%), neutropenia (n=2, 11%), hyperglycemia (n=1, 6%), hypoklemia (n=1, 6%), and anemia (n=1, 6%). Grade 1-2 infections (n=5, 27%) included thrush, sinusitis, and pneumonia with no patients requiring dose delays or hospitalization. Other frequently observed grade 1-2 toxicities were transient hyperglycemia (n=12, 66%), thrombocytopenia (n=11, 61%), reversible infusion reactions (n=9, 50%), and fatigue (n=8, 44%). Human anti-veltuzumab and anti-milatuzumab antibodies, collected pretreatment and day 1 of weeks 4, 12, and 36, have not been detected in any patient. To date, complete responses were observed in 2 patients including one with grade 1-2 follicular NHL (3 prior therapies) who was rituximab-refractory and ultimately underwent allogeneic transplant and one with marginal zone lymphoma (1 prior therapy). Partial responses were observed in 2 patients; one with grade 3 follicular NHL refractory to rituximab with 3 prior therapies including autologous transplant and one patient who had received 5 prior therapies. All responding patients achieved response following induction therapy. Stable disease (SD) was observed in 10 patients including 1 patient with MCL of a median duration of 5.25 months to date (range 2.5-12 months) and 2 patients remain on protocol therapy. Combination therapy with veltuzumab and milatuzumab was well-tolerated in a population of heavily pre-treated patients with relapsed or refractory NHL, 22% having an objective overall response, including rituximab-refractory patients. Enrollment in the phase II study of selected NHL subtypes including indolent NHL and MCL is ongoing.

## NOVEL BIOLOGIC AGENTS FOR TREATMENT OF MCL

In addition to the ongoing evaluation of monoclonal antibody therapy in relapsed or refractory MCL, several targeted biologic agents are undergoing preclinical and clinical evaluation at our institution and others have shown early promise as effective therapeutic agents in MCL.

PCI-32765 is an orally bioavailable inhibitor of Bruton's tyrosine kinase (BTK), which is a key component of the B-cell receptor signaling pathway [53]. It selectively and permanently inhibits BTK, resulting in inhibition of B-cell activation and downstream signaling of the B-cell receptor [54]. Preclinical testing in canine B-cell NHL resulted in objective responses [54]. Preclinical evaluation in chronic lymphocytic leukemia (CLL) demonstrated that *in vitro*, PCI-32765 induced apoptosis in CLL cells via a caspase-dependent mechanism as well as inhibited activation-induced CLL cell proliferation [55]. PCI-32765 demonstrated clinical responses with minimal toxicity in the phase I study in relapsed and refractory B-cell malignancies [56]. The objective RR was 43% for 47 patients enrolled. A total of four patients with MCL were enrolled and three of four achieved an objective response, with all three patients remaining on study for greater than 6 months. Grade 3 or higher toxicities occurred in 19% of patients and included grade 3 neutropenia. The preliminary results of the ongoing phase II study of PCI-32765 were recently reported [57]. A total of 48 patients with MCL were enrolled, in cohorts of bortezomib-naïve and bortezomib-exposed, with 24 patients evaluable for response. The median age was 67 years (range 62-72) and the median number of prior therapies was 2 (range 1-5) including 5 patients (13%) who had undergone prior stem cell transplant. Patients received continuous daily dosing of 560 mg orally of PCI-32765. The ORR for both cohorts was 67% (16/24); ORR is 58% (7/12) in the bortezomib-naive cohort and 75% (9/12) in the bortezomib-exposed cohort. Treatment was well-tolerated with the most frequent grade 1-2 toxicities including fatigue, diarrhea, and nausea. PCI-32765 is currently undergoing evaluation both as a single agent and in combination with immuno-chemotherapy in relapsed or refractory MCL (NCT012363910) and B-cell NHL (NCT00849654 and NCT01109069) at our institution and others.

The phosphatidylinositol 3-kinase (PI3K)/AKT pathway is central to the survival of several different B-cell NHL histologies including MCL and therefore may represent an attractive therapeutic target [58-60]. AKT is a serine threonine kinase that regulates cell survival, proliferation, and apoptosis, in NHL [61]. Constitutive activation of AKT has been shown to be essential to the pathogenesis and survival of MCL [21, 62, 63]. *In vitro* testing of MCL cell lines with AKT inhibitors including LY294002 and wortmannin resulted in apoptosis via a caspase-dependent mechanism [21, 64]. However a phase II testing of ezastaurin, an oral serine/threonine kinase inhibitor which suppresses signaling through the PI3K/AKT pathway, in relapsed and refractory MCL resulted in modest clinical activity [65].

CAL-101 is an oral p110δ selective PI3K inhibitor [66]. Inhibition of the PI3K pathway with CAL-101 in a variety of hematologic malignancies *in vitro* resulted in apoptosis associated with a decrease in phosphorylated AKT (p-AKT) levels and other downstream targets such as p-S6 and GSk3-β [66]. In another recent preclinical evaluation, CAL-101 treatment resulted in caspase-dependent apoptosis of CLL cells [67]. Importantly, CAL-101 treatment did not result in apoptosis of normal T-cell or NK-cells, and did not affect antibody-dependent cellular cytotoxicity when combined with mAbs such as rituximab. Additionally, CAL-101 inhibited the production of proinflammatory/prosurvival cytokines by T-cells and NK-cells including IL-6, IL-10, TNF-α, and INF-γ, suggesting that blocking production of these cytokines *in vivo* would potentially have the effect of antagonizing their survival effects on CLL cells. Furthermore, the investigators postulate that CAL-101 may abrogate infusional toxicity seen with mAb therapy such as rituximab through decreased production of these cytokines.

The promising preclinical data supported clinical development of this agent. In the ongoing phase I clinical trial with CAL-101 (NCT00710528, NCT01090414) in patients with relapsed and refractory hematologic malignancies, responses were observed at all dose levels. At the time of last reporting, 55 patients with B-cell NHL had enrolled (28 patients had indolent NHL: follicular lymphoma n=15, small lymphocytic lymphoma n=6, Waldenstrom's macroglobulinemia n=4, marginal zone lymphoma n=3; 27 had aggressive NHL: MCL (n=18), diffuse large B-cell lymphoma (n=9). Approximately half of the patients had refractory disease with a median of 5 prior regimens. Dose levels ranged from 50 mg to 350 mg orally twice daily. The primary observed dose limiting toxicities were reversible liver function test abnormalities. Hematologic toxicity was infrequent. The overall response rate was 62% (10 out of the 16 evaluable MCL patients), with a median duration of response of 3 months (range 1-8) [68].

Given the central role of the PI3K/AKT pathway in NHL [69-71], downstream targets such as mTOR represent another promising therapeutic target [21]. Several mTOR inhibitors have been evaluated in relapsed and refractory MCL, and temsirolimus and everolimus have been studied most extensively [72-76]. The initial phase II study of temsirolimus in relapsed and refractory mantle cell lymphoma utilized a dose of 250 mg intravenously, administered weekly [73]. The ORR was 34% with a median time to progression of 6.5 months. The primary toxicities observed were myelosuppression, mucositis, fatigue, hyperglycemia, infections, and hypertriglyceridemia [73]. Due to the observed toxicities and frequent need for dose reductions, a phase II study of 25 mg weekly of temsirolimus was performed. The ORR was 41% with a median time to progression of 6 months. The authors concluded that the lower dose retained similar activity but was better tolerated with less myelosuppression [74]. A phase III open label study of temsirolimus administered on a scheduled of 175 mg weekly followed by either 75 mg or 25 mg weekly was compared to investigator's choice in patients with relapsed or refractory MCL. The median PFS was 4.8 months, 3.4 months, and 1.9 months for temsirolimus 175/75 mg, 175/25 mg, and investigator's choice, respectively. The ORR for temsiroliumus 175/75 mg was 22%, and the primary adverse events were asthenia and hematologic toxicities [75]. In a promising phase II study, temsirolimus was combined with rituximab in patients with relapsed and refractory MCL. The dosing schedule included temsirolimus 25 mg weekly and rituximab 375 mg/m2 weekly for 4 weeks and then every other month for up to 12 cycles. The ORR was 59%; for rituximab-sensitive patients the ORR was 63%, and for rituximab-refractory patients the ORR was 52% [77]. Everolimus is an orally bioavailable mTOR inhibitor which demonstrated anti-tumor activity in several histologic subtypes of NHL including MCL [73]. A phase II study of everolimus 10 mg daily in patients with relapsed and refractory MCL demonstrated an ORR of 20% with an additional 49% patients experiencing stable disease. Median progression free survival was 5.5 months. The primary observed toxicities were hematologic in nature [76]. Similar findings were observed in a phase II study of everolimus in patients with relapsed aggressive lymphomas where the ORR was 30% and 6 of 19 (32%) patients with MCL had an objective response [73]. Currently, studies are ongoing evaluating this class of drugs in MCL as single agents, in combination with chemotherapy, and in combination with other targeted therapies.

In summary, MCL is an aggressive B-cell malignancy which is incurable with standard therapies. While the response rate to initial therapy is high, patients invariably relapse, with a tendency toward lower response rates and shorter duration of remissions with subsequent therapies. Our group has demonstrated preclinical *in vitro* and *in vivo* activity in MCL with the combination of milatuzumab and anti-CD20 mAbs and milatuzumab with FTY720. Currently, phase I/II testing of the combination milatuzumab and veltuzumab in patients with relapsed and refractory B-cell NHL is ongoing. Initial toxicity data from phase I study has been primarily related to reversible infusion reactions. Preliminary activity data from the first patients enrolled in the phase I study has been encouraging with four of eighteen heavily pretreated patients responding, although several different NHL histology were included. Other potentially effective novel therapies for MCL actively undergoing investigation include the Bruton's tyrosine kinase inhibitor, PCI-32765, mTOR inhibitors such as temsirolimus and everolimus, and the PI3 kinase inhibitor, CAL-101. Future directions in the treatment of MCL include combinations of mAbs, targeted biologic agents, and cytotoxic chemotherapy.
